# A sequential learning model with GNN for EEG-EMG-based stroke rehabilitation BCI

**DOI:** 10.3389/fnins.2023.1125230

**Published:** 2023-04-17

**Authors:** Haoyang Li, Hongfei Ji, Jian Yu, Jie Li, Lingjing Jin, Lingyu Liu, Zhongfei Bai, Chen Ye

**Affiliations:** ^1^Translational Research Center, Shanghai Yangzhi Rehabilitation Hospital (Shanghai Sunshine Rehabilitation Center), School of Electronic and Information Engineering, Tongji University, Shanghai, China; ^2^Department of Neurology and Neurological Rehabilitation, Shanghai Disabled Person's Federation Key Laboratory of Intelligent Rehabilitation Assistive Devices and Technologies, Yangzhi Rehabilitation Hospital (Shanghai Sunshine Rehabilitation Center), School of Medicine, Tongji University, Shanghai, China; ^3^Neurotoxin Research Center of Key Laboratory of Spine and Spinal Cord Injury Repair and Regeneration of Ministry of Education, Neurological Department of Tongji Hospital, School of Medicine, Tongji University, Shanghai, China

**Keywords:** brain-computer interfaces, neurofeedback, graph neural network, stroke rehabilitation, corticomuscular coherence, sequential learning

## Abstract

**Introduction:**

Brain-computer interfaces (BCIs) have the potential in providing neurofeedback for stroke patients to improve motor rehabilitation. However, current BCIs often only detect general motor intentions and lack the precise information needed for complex movement execution, mainly due to insufficient movement execution features in EEG signals.

**Methods:**

This paper presents a sequential learning model incorporating a Graph Isomorphic Network (GIN) that processes a sequence of graph-structured data derived from EEG and EMG signals. Movement data are divided into sub-actions and predicted separately by the model, generating a sequential motor encoding that reflects the sequential features of the movements. Through time-based ensemble learning, the proposed method achieves more accurate prediction results and execution quality scores for each movement.

**Results:**

A classification accuracy of 88.89% is achieved on an EEG-EMG synchronized dataset for push and pull movements, significantly outperforming the benchmark method's performance of 73.23%.

**Discussion:**

This approach can be used to develop a hybrid EEG-EMG brain-computer interface to provide patients with more accurate neural feedback to aid their recovery.

## 1. Introduction

### 1.1. Sequential motor rehabilitation

Motor imagery (MI) is the mental process of imagining movement in the absence of physical movement (Aggarwal and Chugh, 2019). Brain-computer interfaces (BCIs) based on motor imagery are widely used in rehabilitation training for stroke patients (McAvinue and Robertson, 2008). By using MI, patients can be trained to gain control over their brain signals, allowing them to activate devices that assist with movement. This training approach is believed to enhance sensory inputs, leading to brain plasticity that improves motor function (Hwang et al., 2009). The feasibility of this rehabilitation strategy has been demonstrated by using electroencephalography (EEG) analysis to capture a patient's motor intention and enable them to control an external device. Compared to traditional motor rehabilitation, EEG-based MI approaches allow for active training motivated by the subject's autonomous intention and have been shown to produce better rehabilitation outcomes for patients after the recovery plateau of stroke (Young et al., 2014).

Movements are composed of multiple sub-actions or action units, and the ability to capture the brain's sequential motor intention can provide patients with more refined feedback for better recovery (Xue et al., 2021). However, due to impaired motor function, stroke patients may perform movements in a compensatory manner, resulting in movement patterns that differ from those of a healthy individual (Alankus and Kelleher, 2012). Therefore, a method is needed to assess movements at a finer level of intensity to determine whether they are being performed correctly at each stage.

Currently, it is difficult to identify complex motor intentions from EEG signals (Jerbi et al., 2011). As a result, many BCI systems currently only support a few fixed and simple movements. Electromyography (EMG) is the most direct signal for assessing and perceiving motor execution and has been used in motor rehabilitation training and assessment (Balasubramanian et al., 2018). Deep neural networks based on EMG signals can be used for some more complex tasks such as gait recognition with a high recognition rate (Yao et al., 2021). Recently, it has been proposed that a hybrid BCI can be created by introducing EMG signals into an EEG-based BCI. By combining EEG and EMG analysis, both the motor intention of the brain and the actual execution can be obtained. The high spatio-temporal resolution of EMG also allows different stages of motor intention to be analyzed separately.

### 1.2. EEG-EMG-based Hybrid BCIs

Current brain-computer interfaces (BCIs) based on electroencephalography (EEG) and electromyography (EMG) are relatively simple. Many studies use EEG to detect motor intention and then use EMG to analyze the actual execution of the movements (Sarasola-Sanz et al., 2017; Ruhunage et al., 2019). Some studies have integrated EEG and EMG models at the model level to improve classification accuracy (Zhang et al., 2019). Others use EMG to remove motion artifacts from EEG to improve signal quality for better classification (Hooda et al., 2020). For motor execution classification of healthy individuals, EMG is sufficient to provide good results, but for stroke patients with motor impairment, the signal quality of EMG may be weaker and thus EEG is needed as an aid.

A rehabilitation system based on EEG and EMG has different implications for different types of stroke patients. For patients with poor motor control, a system based on EEG may be more beneficial as it can detect motor intention without requiring physical movement. For patients with better motor control, a system based on EMG may be more beneficial as it can provide detailed analysis of movement execution (Cesqui et al., 2013). By using both signals, a more comprehensive rehabilitation plan can be tailored to the specific needs of each patient.

In the field of stroke rehabilitation, corticomuscular coherence (CMC) is a useful tool for describing changes in central nervous system activity (Liu et al., 2019). CMC is considered a potential biomarker of motor deficits after stroke, capable of quantifying recovery and potentially indicating the cortical areas involved in functional recovery (Lattari et al., 2010; Krauth et al., 2019). However, many CMC-based BCIs have not been able to achieve good results (Chowdhury et al., 2019), likely because CMCs tend to focus only on the overall correlation between EEG and EMG, rather than on a more fine-grained level of connectivity. One study correlated EEG-EMG for four motor tasks and found that coherence was higher in the contralateral brain cortex than in the ipsilateral motor cortex (Tun et al., 2021). One study used channel correlations to create networks between EEG and EMG to analyze recovery in stroke patients (Tan et al., 2022).

### 1.3. Graph neural networks for BCIs

The connections between neurons, or brain networks, have been studied extensively. Some cognitive patterns can be explored by analyzing the connectivity between different brain regions. Structural connectivity is often obtained by studying the anatomy of the brain, while functional connectivity is often obtained by correlation analysis of signals such as EEG and fNIRS (Bullmore and Sporns, 2009). Some studies have used dynamic functional brain networks to extract features in the EEG signal for epilepsy prediction (Li et al., 2021a) and MI (Zhang et al., 2021) with high accuracy and robustness. Such analysis and brain-muscle coupling are essentially equivalent. Fine-grained corticomuscular coherence (CMC) allows EMG to be incorporated into brain networks, allowing both signals to be analyzed together. This can be particularly useful for studying motor intention detection in the context of stroke rehabilitation.

Graph Neural Networks (GNNs) aim to build neural networks using graph theory to process data in the graph domain. GNNs have seen rapid development in recent years and have been increasingly used in the field of BCIs with good results (Tang et al., 2021). With this new tool, GNN models can be used to analyze complex graph structure data that is difficult to analyze using traditional methods. Researchers have proposed various GNN models for EEG-based BCIs that project electrodes onto nodes of a graph, where node features are represented as samples of EEG channels collected during the experiment. These nodes can be connected by edges according to a flexible strategy developed by neuroscientists. These networks have achieved better classification results than commonly used deep learning models. One study built a graph convolutional network for the motion imagery task, which achieved excellent results on publicly available datasets and was able to provide visual features for motion imagery (Sun et al., 2021). Another study used mutual information as edge weights of the graph, combined with a spatio-temporal graph convolutional network, and achieved better classification accuracy than current state-of-the-art methods (Li et al., 2021b). However, there is still a gap in the classification of correlation maps between EMG and EEG.

### 1.4. Main contributions

The main contribution of this paper is the introduction of GNNs into the classification of multimodal physiological data (EEG-EMG), which captures both spatial and temporal information and generates a sequential coding of a movement. Furthermore, this paper selects SPMI for graph construction and GIN for GNN backbone model, achieving significant results beyond the baseline methods, and also compares and discusses other GNN models and graph construction methods. Through time-based ensemble learning, the method proposed in this paper can be used to assess each movement, which is useful for stroke rehabilitation. In the future, our proposed model can be used to achieve better classification, more interpretable evaluation of complex actions, and more detailed neurofeedback.

## 2. Materials and methods

In this section, a general framework for this sequential learning model based on graph neural networks (GNNs) for EEG-EMG data is proposed. The construction of graphs from the data and the use of the GNN model to classify them are described. Additionally, a time-based ensemble learning approach for continuous motion recognition and movement quality assessment with split data trials is presented.

### 2.1. Model framework

**Figure 1:**
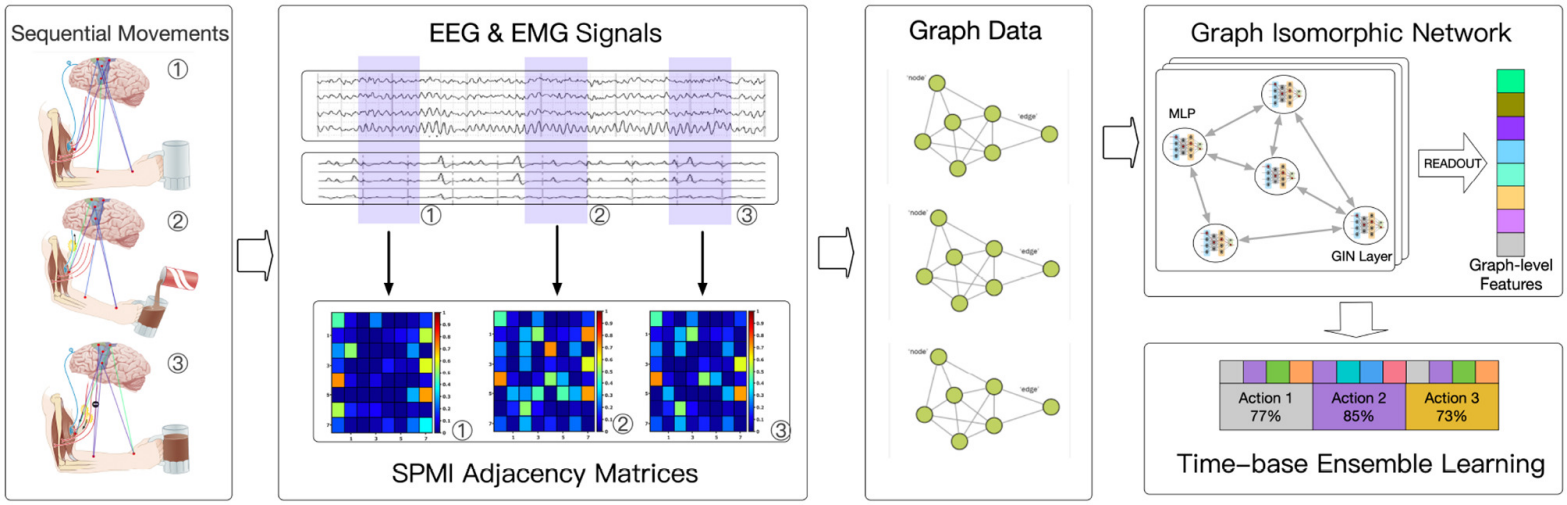
Model framework.

Figure 1 shows the general framework of the model proposed in this paper. The process begins by acquiring synchronous EEG and EMG signals and dividing them into small segments based on time. Pairwise mutual information is then computed to generate graph structure data. This data is fed into a graph neural network, which is used to learn the action units represented by each data segment. Finally, sequences of these sub-actions are smoothed to determine the complex movements over a given time period and to assess their quality.

Data from 32 EEG channels and 8 EMG channels were used in the implementation. The data was sliced once per second, and the signal from each channel was processed using a multitaper to obtain Power Spectral Density (PSD). PSD was then used to construct the features of each channel. Connectivity between each channel was calculated using normalized pairwise mutual information (SPMI). Graph Isomorphic Network (GIN) (Xu et al., 2018) was used for the classification of graph-structured data. Each complex movement was sliced into 1-s segments, and the model learned which kinds of movement each segment belonged to and when it occurred. Finally, a time-based ensemble learning approach was performed to combine the outputs of the model and determine the complex movement for each period and assess its quality.

### 2.2. EEG-EMG heterogeneous graphs construction

A graph is an ordered pair *G* = {*V, E, A*}, where V represents the channel set |*V*| = *N*, and *E* represents the connection between the channels. *V* = {*V*_1_, *V*_2_, ..., *V*_*N*_}, each $V_{i}\in R^{1d}$ represents the feature of the *i* th channel where *d* represents the dimension of the feature. *A*∈*R*^*NN*^ is the adjacency matrix, which represents the connection relationship between the two channels in *V*. Among them, *A*_*ij*_ represents the importance of the connection between the *i* th channel and the *j* th channel.

The node feature is obtained by using multitaper to compute the power spectral density (PSD) and provide frequency domain information. Similar to the traditional physiological signal processing method, the signals in different frequency bands are focused separately. The percentage of power in different frequency bands to the total power is used as the node feature, and the numbering of electrodes is added to the node features as a location code, to better learn the structural information in the graph. The focused frequency bands are Delta, Theta, Alpha, Beta, and Low Gamma, so each node will have a feature of length 7, including the percentage of total power for each of the five bands, the total power, and the numbering of the electrode.

Standardized Permutation Mutual Information (SPMI) (Afshani et al., 2019) is used to construct edges in graphs. SPMI is capable of detecting both linear and non-linear statistical dependence, and has been widely used in neurological studies as a measure of communication between cortical areas. The distribution of SPMI is smooth and continuous between EEG and EEG channels, EEG and EMG channels, and EMG and EMG channels. Therefore, SPMI is a suitable coherence metric for graph construction. For a EEG or EMG signal *X*= *x*(*t*)(*t* = 1, 2, …, *N*), the set of embedding vectors is *x*_*i*_(*i* = 1, 2, …, *N*−(*n*−1)τ) according to


(1) $$\begin{array}{l} x_{i}=\left[x\left(i\right),x\left(i+\tau \right),\ldots x\left(i+\left(n-1\right)\tau \right)\right] \end{array}$$

where *n* is embedding dimension and τ is time lag. The embedding vectors are the fragments of EEG or EMG signals. The embedding vector's ordinal pattern was determined by ranking the signal value. Therefore, each signal value can be permutated into *n*! kinds of ordinal pattern possibilities. Then, the Permutation Entropy (PE) of a signal *X* is defined as the following formula (Bandt and Pompe, 2002):


(2) $$\begin{array}{l} \text{P}{\text{E}}_{X}=-\overset{n!}{\underset{j}{\sum }}P_{X}\left(j\right)\log\left(P_{X}\left(j\right)\right) \end{array}$$

*P*_*X*_(*j*) is the empirical probability of *j* th ordinal pattern. Then, the joint *PE* of *X* and *Y* is defined as the following formula (Afshani et al., 2019):


(3) $$\begin{array}{l} \text{P}{\text{E}}_{X,Y}=-\overset{n!}{\underset{j}{\sum }}\overset{n!}{\underset{k}{\sum }}P_{X,Y}\left(j,k\right)\log\left(P_{X,Y}\left(j,k\right)\right) \end{array}$$

*P*_*X, Y*_(*j, k*) is the joint probability of permutation of *X* and *Y*. Finally, *SPMI* between two time series X and Y can be calculated as the following formula (Afshani et al., 2019):


(4) $$\begin{array}{l} \text{SPM}{\text{I}}_{X,Y}=\frac{\text{P}{\text{E}}_{X}+\text{P}{\text{E}}_{Y}-\text{P}{\text{E}}_{X,Y}}{\text{P}{\text{E}}_{X,Y}}. \end{array}$$

The range of SPMI is between 0 and 1, which allows obtaining a standardized degree of correlation between each channel. By connecting the channels with a high degree of association according to a threshold, the connectivity between channels, which is the edge information of the graph, can be obtained.

The graph used in this study contains both EEG and EMG data, and the difference between EEG and EMG data makes the graph containing both data and the relationship between them a heterogeneous graph. After separate preprocessing, the preprocessed EEG and EMG data are processed into graph data in the same way. This is to allow a general GNN (e.g., GIN) to handle EEG-EMG synchronization data as well. the GNN will treat the graph data as general graphs. The method of edge concatenation is also consistent for inter-EEG and inter-EMG connections, with SPMI serving as a measure of mutual information between nodes. For connections between EEG and EMG channels, the SPMI can be considered as a measure of corticomuscular coupling (CMC), providing insight into complex brain-muscle interactions. This allows the use of a graph neural network to analyze the data and potentially improve the classification of motor intentions in stroke rehabilitation.

### 2.3. Graph isomorphic network

The Graph Isomorphic Network (GIN) is used to process the graph structure data of EEG-EMG. The GIN model is described by the following equation:


(5) $$x_{i}^{\prime }=h_{\Theta }\left((1+\epsilon )\cdot \;x_{i}+\sum_{j\in N(i)}x_{j}\right)$$

where *h* is a multilayer perceptron (MLP) for fitting the combination of functions. In this study, the network is a two-layer neural network with batch normalization. The variable *x*_*i*_ is the node feature and *N*(*i*) is the neighbor set of node *i*. The variable ϵ is a learnable irrational number parameter.

Node embeddings learned by GIN can be directly used for tasks such as node classification and link prediction. For graph classification tasks, the following readout function is performed. It can transform given embeddings of individual nodes to the embedding of the whole graph as the followed formula (Xu et al., 2018):


(6) $$\begin{array}{l} h_{G}=\text{CONCAT}\left(\text{READOUT}\left(\left\{h_{v}^{\left(k\right)}|v\in G\right\}\right)|k=0,1,\ldots ,K\right) \end{array}$$

Where *h*_*G*_ is the graph embedding, *READOUT* is the readout function that applies a linear transformation to all node embeddings, *CONCAT* concatenates the output of *READOUT* function, and *k* is the number of iterations of the GIN model. As the number of iterations increases, the node representations corresponding to subtree structures in the graph-level readout become more refined and global. A sufficient number of iterations is crucial for achieving good discriminative power. However, features from earlier iterations may sometimes generalize better. To account for all structural information, information from all depths or iterations of the model is used. This allows the GIN to effectively classify complex motor intentions and improve rehabilitation outcomes for stroke patients.

### 2.4. Time-based ensemble learning approach

The input to the GNN model is a graph-structured representation of EEG and EMG data over time, and the output is a label representing a specific movement. However, this model ignores the high temporal resolution of EMG and EEG and does not allow for the assessment of complex movements. To capture the time domain features of a movement and to assess its quality, the data of each trial is segmented and fed into the GNN model separately, resulting in labels for the corresponding sub-actions. Each sub-action label consists of two parts: the movement to which the sub-action belongs and the segment of that movement to which the sub-action belongs. By connecting the predicted labels of the sub-actions, a sequence of predicted labels can be obtained. By analyzing this sequence, the movement can be classified and its score calculated.

Suppose the sequence of predicted sub-actions is M[n], where M[i] represents the prediction of the *i* th sub-action given by the model. The actual label is G[n] and G[i] represents the actual label of the *i* th sub-action. The score of the movement is then calculated as:


(7) $$score_{G_{i}}=\frac{\sum_{i=1}^{n}similarity(G_{i},M_{i})}{n}$$

where the similarity is defined as:


(8) $$similarity(a,b)=\{\begin{array}{ll} 0 & \text{a and b are from different movement}\\ 1-\frac{\left|a-b\right|}{n} & \text{a and b are from same movement} \end{array}$$

**Figure 2:**
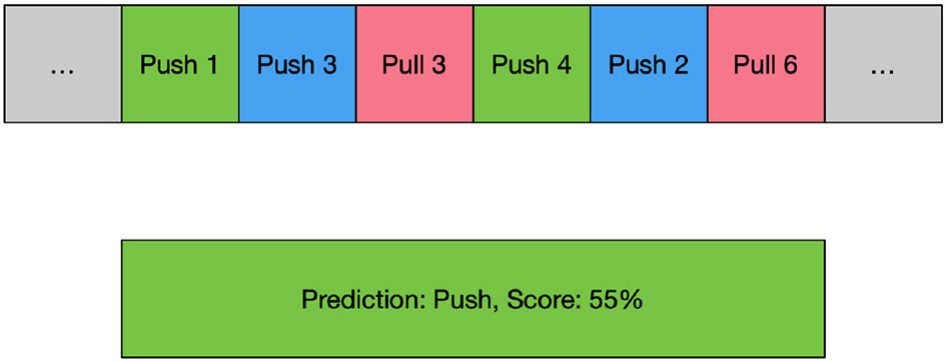
An example of time-based ensemble learning. Green blocks mean the model predicted the movement and the position exactly, blue blocks mean the model predicted the movement correctly but not the same position, and red blocks mean the model predicted a wrong movement.

The score of the predicted label sequence for each movement can be calculated, and the movement with the highest score can be obtained as the predicted output. This improves the overall classification accuracy. Figure 2 shows an example of the process of the ensemble. This is a labeled push action, and the model first divides the movement into six segments to separately predict which segment belongs to which action. Then, the scores of each possible movement label are calculated separately according to equation (7), and the label with the highest score is taken as the overall prediction. In the example in the Figure 2, this movement data is predicted to be a push and has a score of 55.

## 3. Experiments and results

### 3.1. Datasets

**Figure 3:**
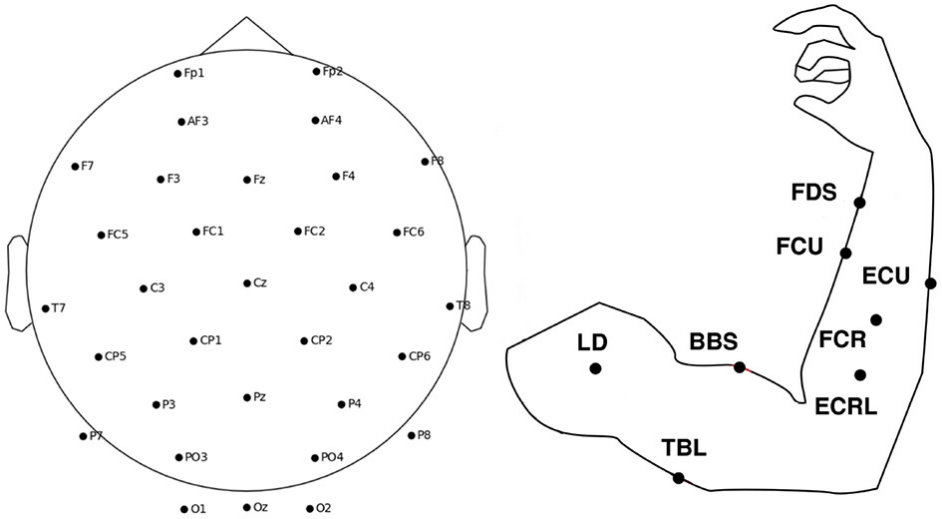
The location of EEG and EMG electrodes.

A public dataset of simultaneous EEG-EMG acquisition (Tan et al., 2022) was used for the experiment. The data consisted of 32 channels of EEG data according to the international 10-10 system. The EEG sampling rate was 500 Hz. Eight EMG electrodes were placed on the arm at the following positions: (a) flexor digitorum superficialis (FDS), (b) flexor carpi ulnaris (FCU), (c) flexor carpi radialis (FCR), (d) extensor carpi ulnaris (ECU), (e) extensor carpi radialis longus (ECRL), (f) biceps brachii short head (BBS), (g) triceps brachii long head (TBL) and (h) lateral deltoid (LD). Figure 3 shows the location of EEG and EMG electrodes. The EMG sampling rate was 1,000 Hz. The dataset collected EEG EMG data from 5 healthy volunteers and 2 stroke patients performing isometric push and pull movements of 3 s duration. The dataset contains data from a total of 516 trials of healthy individuals and 174 trials of stroke patients.

### 3.2. Data preprocessing and graph construction

To prepare the data for analysis, the EMG data were downsampled to 500 Hz and aligned with the EEG data. The EEG signals were band-pass filtered from 2 to 40 Hz and the EMG signals were band-pass filtered from 2 to 100 Hz. Noise was removed using Independent Component Analysis (ICA) with a threshold of 99%. Finally, the epochs were divided according to the time of the action cues and grouped from 0 to 3 s. Many significant signal power changes occur at integral multiples of 0.5 s, such as 1.5 s and 2 s, based on the observed EMG signals. Therefore, each data epoch is divided into six segments of 0.5 s each. The data was divided into training and test sets at a ratio of 4:1. The model obtained from the training data was then evaluated on each subject's test set, resulting in a cross-subject model.

After preprocessing, the segmented EEG-EMG data is used to construct the graph data. The information in the nodes is based on the power spectral density of the data for each channel, while the relationships between the nodes are based on normalized pairwise mutual information.

To extract node features for the graph structure, the Power Spectral Density (PSD) is calculated for each channel's signal using multitapers. The percentage of each band in the total power is then calculated. The specific band divisions are Delta (2–4 Hz), Theta (4–8 Hz), Alpha (8–15 Hz), Beta (15–30 Hz), and Low Gamma (30–40 Hz). These divisions combine physiological significance and experimental results and are slightly different from the usual band divisions. However, the banding has little effect on the results. Total channel power and channel number are also included as node information.

For edge information, the SPMI between pairs of channels is computed with an embedding dimension of 5 and a time delay of 1. The 25% percentile of all SPMIs is then used as the threshold for connecting edges in the graph. This results in a graph with 195 edges for a graph with 40 points. This value was determined experimentally, and the effect of different graph construction methods on the results is analyzed.

### 3.3. Graph feature analysis

**Figure 4:**
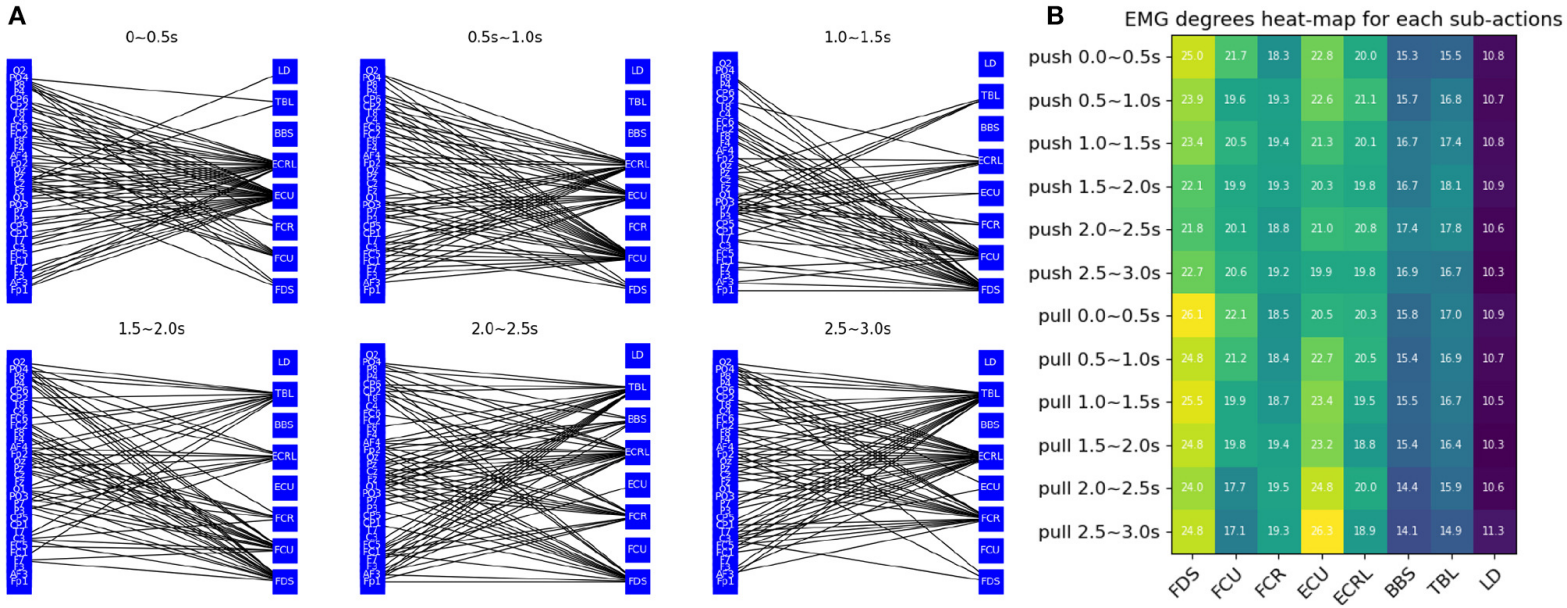
Graph feature analysis **(A)** visualization of the connectivity between EEG and EMG channels **(B)** each labels' EMG degrees heat-map for each sub-actions.

Figure 4A visualizes the connectivity between each channel over time. As time changes, the connectivity of the graph changes in different ways. The figure shows the pushing movement of a typical healthy person (subject 19). It can be seen that different EMG channels have more connections with EEG channels over time. For example, channel ECU and ECRL are connected to more EEG channels in 0 0.5s, which gradually changes to more connections in channel FCU and FDS in the third figure after 0.5s. Then channel FCR and TBL got more connections. Channel BBS and LD don't have many connections during this movement because they are not related to this movement. The change in EMG and EEG connections is essentially giving weight or attention to the node information of the graph to help the model identify important features. This is thought to reflect the sequential behavior of the brain controlling the muscles.

The average degrees of the EMG channel for each sub-action are shown in Figure 4B. A clear change in connectivity can be observed during the different phases of different movements. For example, the degree of the ECU gradually decreases during the push and gradually increases during the pull. The opposite change can be observed in the TBL channel.

### 3.4. Experiments settings

The Graph Isomorphic Network (GIN) model was used in this study to process graph-structured data. The GIN was trained with 300 epochs and a batch size of 128. The multilayer perceptron in each node has an input channel of 7 (the dimension of the node features), 64 hidden channels, and 2 layers of MLP.

The training phase used the Adam optimizer with a learning rate of 0.005. The model was trained with the sum of the cross-entropy loss for sub-actions and whole movements. In the evaluation phase, the average classification accuracy was used as a metric to evaluate the performance of the model.

To evaluate the trained GIN model, it was first used to predict the labels of each sub-action for each trial. The sequence of predicted labels was then used to determine the overall predicted label for the trial, as well as to assess the quality of the movement. The scores for each movement were calculated, and the movement label with the highest score was selected as the overall predicted label. Finally, the predicted labels were compared to the ground truth labels to evaluate the performance of the model.

### 3.5. Classification results

In the experiment, the proposed GIN-based model was trained using data from all individuals and its performance was evaluated using data from each healthy individual and stroke patient, respectively. The performance of the model was compared with two commonly used benchmark methods: CSP+SVM (Ang et al., 2008) and EEGNet (Lawhern et al., 2018). In addition, two other types of GNNs were tested: Graph Attention Network v2 (Brody et al., 2021) and Residual Gated Graph ConvNets (Bresson and Laurent, 2017). All experiments were repeated five times and the average is shown.

Table 1 displays the results of every patient on the approach used in the study and the baseline models. The results showed that the GIN-based model outperformed the benchmark methods. It achieved 88.89% classification accuracy, significantly higher than the 73.23% of CSP+SVM and 67.60% of EEGNet. The poor performance of EEGNet is mainly due to its reliance on a large amount of data, while the proposed method does not require a large amount of data due to its simpler structure. In addition, the higher classification accuracy of the proposed method demonstrates its ability to capture important features and its ease of training. CSP+SVM is a common benchmark method that usually performs well on small datasets. However, in this study, it did not perform as well on the data set, which may be due to the large differences between EEG and EMG data. The other two graph neural networks performed better than CSP+SVM and EEGNet, proving the effectiveness of our graph construction method. Meanwhile, GIN can identify more node features and also better analyze the structural information of the graph, which makes the results of GIN the best performance among the graph neural network methods.

**Table 1:** Every patient's results on our approach and baseline models.

**Methods**	**Health**	**Patients**	**Overall**	**2**	**4**	**14**	**15**	**17**	**18**	**19**
CSP+SVM	75.74%	63.89%	73.23%	69.23%	60.87%	61.90%	76.92%	80.00%	95.65%	75.00%
EEGNet	72.64%	52.77%	67.60%	46.15%	56.52%	57.14%	84.61%	56.00%	95.65%	75.00%
GraphAttentionNet	83.01%	57.86%	77.64%	46.15%	69.57%	71.42%	69.23%	88.00%	91.30%	87.50%
ResGatedGraph	79.24%	70.83%	76.06%	61.53%	69.57%	76.19%	61.53%	88.00%	91.30%	70.83%
Ours	93.96%	73.54%	88.89%	72.30%	74.78%	82.85%	83.07%	99.20%	100.00%	98.33%
Ours(single-subject)	/	/	/	38.46%	82.60%	71.42%	76.92%	92.00%	95.65%	85.83%

The largest result number in each column is in bold.

The experiments in this study were cross-subject, where data from all subjects were used to train the same model during the training process. Compared to the results of the single-subject experiments, where a model was trained for each subject, most subjects had higher accuracy. The single-subject results for Subject 4 were slightly higher than the cross-subject results, probably because stroke patients have their own unique movement patterns. The purpose of the method proposed in this paper is to better assess the movement quality of stroke patients compared to healthy subjects, so a cross-subject experiment is necessary. The GNN-based method proposed in this paper is more suitable for the cross-subject task because it can clearly and separately process data on EEG, EMG, and brain-muscle coupling information.

For stroke patients, the proposed method also outperformed the baseline methods. The poor signal quality of both EEG and EMG data, caused by the patients' weak EMG performance and the effect of brain damage on the EEG, hindered the baseline methods' ability to capture important features. However, the proposed GNN-based method achieved higher classification accuracy due to its use of the EEG-EMG correlation graph, which provides new information to the model. The graph network model also performed consistently on healthy human data, achieving nearly 100% accuracy for three of the subjects.

### 3.6. Ablation experiments

**Figure 5:**
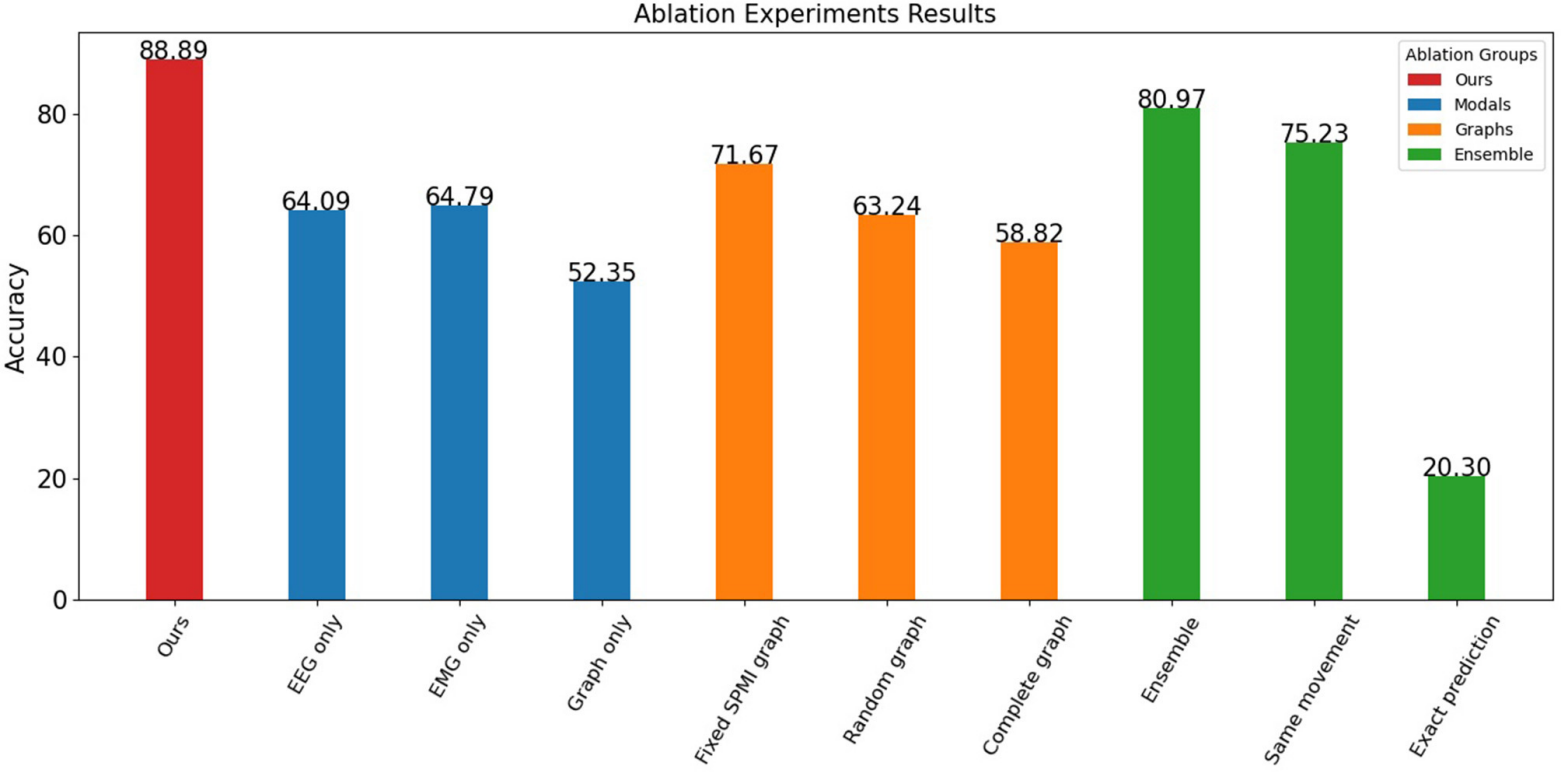
Ablation experiments results.

In this study, several ablation experiments were performed to assess the contributions of different data sources and processing methods to the overall model performance. The results of these experiments are shown in Figure 5.

The results indicate that both EEG and EMG signals contribute to the performance of the model. When only one data source was used, the model achieved an accuracy of 64.09% for EEG and 64.79% for EMG. However, when the graph structure was considered without the node information, the model achieved only 52.35% accuracy, indicating that the graph data based on EEG-EMG coupling mainly reflects the relationship between different node features and contains relatively little information by itself. By incorporating both EEG and EMG data and their relationship through the graph, the model achieved significantly better classification accuracy than using a single data source.

The experiments also showed the contribution of both node and edge information in the graph. The proposed graph construction method was compared with other methods that do not consider SPMI between channels, including random graphs and complete graphs. The accuracy of the random graph (with a 25% probability of connecting edges) and the complete graph was about 60%, while the fixed graph achieved an accuracy of 71.67%. This indicates that the edge information in the graph provides useful information, and that this information is not just a structural, fixed representation, but a feature that changes over time and reflects movements.

The effect of time-based ensemble on the results was also investigated. When the data was not split and classified directly, the accuracy rate reached 80.97%. This shows that the model can still achieve good results without considering the sequential features of the movements. However, when data splitting was added, the model was able to capture more temporal features, resulting in improved accuracy. For the sub-action data, the model was able to identify the corresponding movement with 75.23% accuracy and its exact position within the movement with 20.30% accuracy (12 categories). The mutual information graph used in the proposed method captures better correlation and frequency domain features for each channel, allowing the model to identify the label of a small segment of data with similar accuracy as the entire segment. In addition, by learning the position of sub-actions within the labels, the network can capture the temporal features of the data. This allows the proposed method to achieve high accuracy and assess the quality of each movement.

### 3.7. Movement assessment

**Figure 6:**
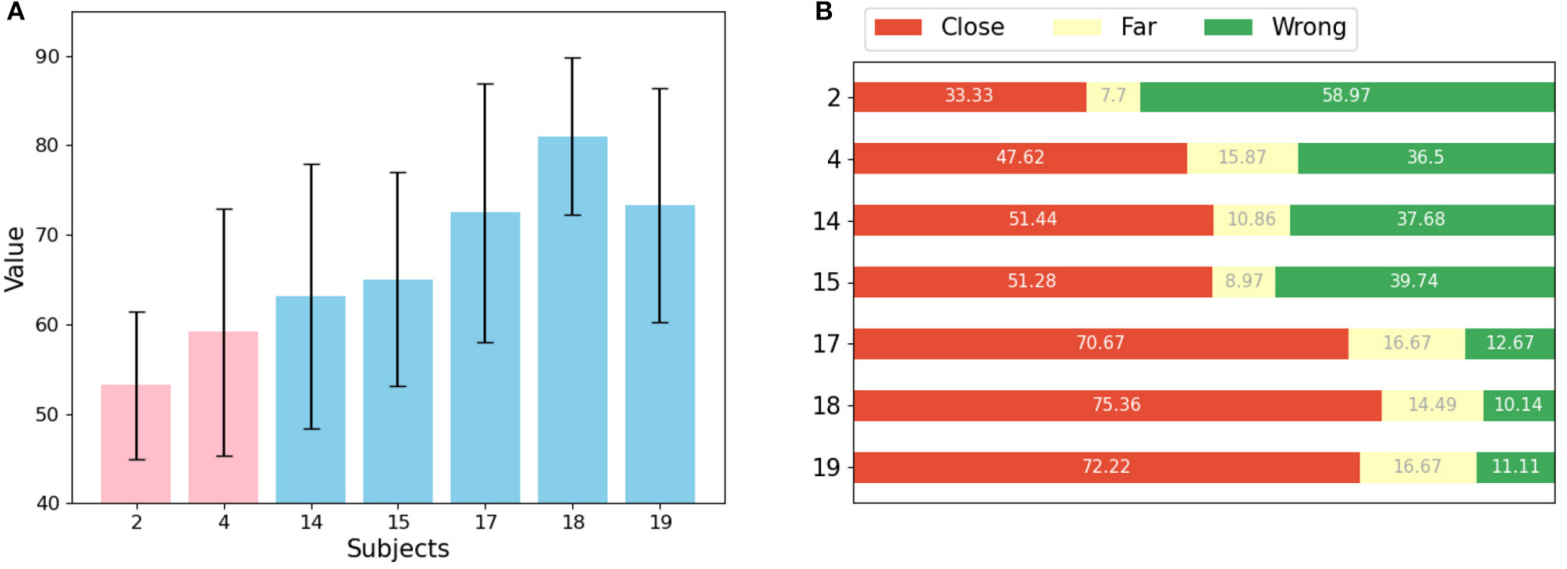
Results of movements assessment **(A)** mean value and std for each patient (pink) and healthy subject (light blue). **(B)** Proportion of predictions for each subject that are correct or close to the label (red), far from the label (yellow), and wrong (green).

A sequential learning approach was used to calculate movement scores for each healthy individual and stroke patient in the dataset. As shown in Figure 6A, the mean scores and variances for healthy individuals are significantly higher and smaller, respectively, indicating that healthy individuals can perform each movement consistently. In contrast, the scores for stroke patients are generally low and have larger variances, likely due to their limited ability to control their movements. In addition, the scores for different patients are at different intervals, indicating that their motor functions are at different stages. Patient 4 was able to perform some of the movements, resulting in a higher score among the stroke patients. Patient 2, on the other hand, had difficulty performing movements independently, resulting in a lower assessment score. This is consistent with the results observed in the EMG data. Independent samples t-tests were used to assess the difference between the scores of the two groups of stroke patients and healthy subjects. The results showed a t-value of -4.88 and a p-value of 2.83e-06. This indicates a significant difference between the movement scores of stroke patients and healthy subjects.

Figure 6B shows the weight of the reasons for low scores for each patient. Two factors can affect the score: if the segmentation belongs to a different movement than the label, or if it belongs to the same movement but appears in a different location. The prediction results of the sub-actions were classified into three categories: close, meaning that the absolute value of the distance between the prediction result and the label is less than or equal to 3, far, meaning that the absolute value of the prediction result and the label is greater than 3, and wrong, meaning that the prediction result and the label belong to different moves. Each sub-action represents some muscle stretch or contraction in time, and since these actions are equally segmented from a full movement, they have no explicit semantic information. It can be observed that the features of each segment are basically similar.

Our results showed that for healthy individuals, most of the data corresponding to sub-actions were accurately or closely predicted by the model. However, for stroke patients, most of the data for sub-actions was predicted incorrectly. The model struggled to accurately predict data for more distant sub-actions of the same movement for all subjects. This is likely because adjacent sub-actions are more similar to each other, making it easier to predict the data of one sub-action as the adjacent sub-action. Since stroke patients often perform movements differently, evaluating sub-actions separately may lead to a more accurate assessment of a movement. However, accurately determining which sub-action a movement belongs to and distinguishing between sub-actions can be challenging for stroke patients, so calculating scores based on the distance between the predicted and true labels can provide a more convincing evaluation of their movements.

### 3.8. Model performance

The proposed model in this paper is designed to be lightweight, making it ideal for use in hospitals and homes for rehabilitation purposes. The training time for 300 epochs takes an average of 4.27 h on a laptop with a quad-core Intel Core i5 processor. The performance bottleneck is mainly in the calculation of SPMI, which has a high computational complexity. However, in practical rehabilitation applications, the model can be quickly retrained with the fully preprocessed data in a few minutes after incremental preprocessing of the new data.

To deploy the model proposed in this paper in a rehabilitation system, it is only necessary to first build a graph of the input data and then predict it with the preloaded model. This process is similar to other common classification models, making it easy to integrate into existing systems. The prediction process takes less than 3 s, providing users with soft real-time feedback. In a practical application, offline computation of the brain-muscle connectivity map for a specific user can be considered to achieve better real-time performance at the expense of some accuracy. The results of ablation experiments show that the model's accuracy is acceptable with fixed maps computed using the SPMI.

Currently, a rehabilitation system based on this model is under development. In the future, it has the potential to be integrated with hardware that provides more detailed feedback, such as functional electrical stimulation and advanced mechanical gloves with increased degrees of freedom.

## 4. Discussion

The model proposed in this paper has potential for further development in two areas.

First, a more robust definition and evaluation of sub-actions is needed. The sub-actions in this paper are simply a complex movement equally divided by time, which allows the capture of action characteristics at a finer granularity. However, the interpretation of these sub-actions is weak. If semantic information can be obtained for each sub-action through the use of kinematic principles or unsupervised learning-based methods, the model may perform better. In addition, the evaluation of actions can be improved by considering the similarity of different sub-actions.

Second, the output of the model can be used to provide more effective feedback to patients. Currently, the model outputs a sequence of sub-actions from which predicted labels and scores can be obtained for the entire action. However, EMG and EEG-based brain-computer interfaces are able to simultaneously capture a user's motor intention and motor execution. This allows us to learn the gap between a user's intended movement and its actual execution. With this information, appropriate compensation can be provided to help the patient perform the correct movement. With this precise feedback, patients can gain a better understanding of their rehabilitation process and gradually become less dependent on exoskeletons.

## 5. Conclusion

In this paper, a novel sequential learning model based on graph neural networks has been proposed for use in EEG-EMG brain-computer interfaces to support the rehabilitation of motor function in stroke patients. Using a publicly available dataset, our method achieves a classification accuracy of 88.89%, significantly outperforming the benchmark method (73.23%) and providing an interpretable movement assessment for each movement. This model has the potential to provide sequential compensatory feedback to patients, allowing them to receive better feedback on the quality of their movements, and may be effective in helping them rebuild their neural circuitry. This technology is currently being used to develop a stroke rehabilitation system to aid in the recovery of hand function in stroke patients.

## Data availability statement

Publicly available datasets were analyzed in this study. This data can be found at: https://github.com/CM-connectivity/CM-graph.

## Author contributions

HL: carried out experiment and writing. HJ, JY, and JL: designed the overall framework. LJ, LL, ZB, and CY: methodological guidance and formal analysis. All authors contributed to the article and approved the submitted version.
